# 0329. Beneficial effects of azithromycin combined with ceftazidime without activity against P. aeruginosa in a murine sepsis model of peritonitis by Pseudomonas aeruginosa

**DOI:** 10.1186/2197-425X-2-S1-P18

**Published:** 2014-09-26

**Authors:** ME Pachón-Ibañez, A Díaz-Martín, J Dominguez-Herrera, G Labrador, Y Smani, J Pachón, J Garnacho-Montero

**Affiliations:** UCEIMP/IBIS-University Hospital Virgen del Rocío, University of Seville, Seville, Spain; University Hospital Virgen del Rocio, Intensive Care Unit, Seville, Spain

## Introduction

Despite a proper management, the mortality of patients with severe sepsis and septic shock is very high. During sepsis, an uncontrolled cascade of inflammatory mediators is triggered, leading to tissue damage that can cause death. Macrolides, apart from their antibiotic properties, are capable of modulating inflammatory response. Macrolides inhibit the production and secretion of pro-inflammatory cytokines (IL-1, IL-6, IL-8 and TNF-α) levels and increase anti-inflammatory cytokines such as IL-10.

## Objectives

To demonstrate whether the use of azithromycin (AZM) in a murine sepsis model of *P. aeruginosa* increases ceftazidime (CFZ) efficacy.

## Methods

MIC/MBC and bactericidal activity (time-killed curves using 1MIC) were performed using four clinical isolates of *P. aeruginosa*. The bactericidal activity on Pa4, chosen for the *in vivo* study, was carried out too at the maximum plasma concentration (Cmax) in mice. Pharmacokinetic/pharmacodynamic (PK/PD) parameters of CFZ and AZM were calculated (HPLC-MS/MS). The activity of each antimicrobial agent and its combination was assessed in a murine peritonitis model (n=15), using the minimum lethal dose (MLD) of Pa4 as inoculum. Treatment were: 100 mg/kg/ip/12h for CFZ and 30 mg/kg/ip/24h for AZM, during 72 hours; a group of 15 untreated mice were used as control (CON). CFU/g spleen, mortality and blood cultures were compared.

## Results

The MIC/MBC (mg/L) ranges of CFZ were 2-4/2-32 and for AZM 64/128->128. In time-kill curves no bactericidal activity was observed with any of the strains at concentration of 1MIC. At concentration of Cmax, CFZ+AZM reached bactericidal activity, but not synergy was found. PK/PD results are shown in table 1. In the animal model (table 2), CFZ and CFZ+AZM were better than CON and AZM, and the combination better than the CFZ and AZM (p< 0.001, ANOVA, post hoc tests) respect to the CFU/g spleen and the blood cultures. No differences were found in the mortality rates (chi-square test). In the survival analysis, CFZ+AZM delayed the mortality compared with the other groups (p< 0.001, Kaplan-Meier).

**Table 1:** PK/PD results

		CFZ	AZM
**PK**	**Cmax (µg/mL)**	107.14	4.57
**PK**	**Tmax (h)**	1.08	2.14
**PK**	AUC0-∞ (µg*h/L)	126.83	3.81
**PD**	**Cmax/MIC**	107.14	0.07
**PD**	**T1/2/MIC**	1.08	0.02
**PD**	**AUC0-∞/MIC**	126.83	1.98

**Table 2:** In vivo studies

	CON	CFZ	AZM	CFZ+AZM
Log UFC/g Spleen (Mean ± SD)	8.40 ± 0.32	6.57 ± 0.61	7.58 ± 0.31	4.63 ± 0.77
Positive blood culture (%)	100	47	100	0
Exitus (%)	100	100	100	100

## Conclusions

The combination of AZM with CFZ increases bacterial clearance from spleen and blood and delays the time to mortality in a murine sepsis model by *P. aeruginosa*.

